# Reduced inflammation in expanding populations of a neotropical bird species

**DOI:** 10.1002/ece3.2486

**Published:** 2016-09-28

**Authors:** Juliette Bailly, Stéphane Garnier, Aurélie Khimoun, Emilie Arnoux, Cyril Eraud, Jean‐Yves Goret, Thomas Luglia, Philippe Gaucher, Bruno Faivre

**Affiliations:** ^1^ BioGéoSciences UMR CNRS 6282 Univ Bourgogne Franche‐Comté Dijon France; ^2^ Office National de la Chasse et de la Faune Sauvage CNERA Avifaune migratrice Villiers en Bois France; ^3^ UMR Ecologie des Forêts de Guyane Kourou Cedex Guyane Française France; ^4^ Groupe d'Etude et de Protection des Oiseaux de Guyane Cayenne Guyane Française France; ^5^ CNRS en Guyane Résidence Le Relais Cayenne Guyane Française France

**Keywords:** biological invasions, ecological immunology, enemy release hypothesis, evolution of increased competitive ability hypothesis, French Guiana, haptoglobin, West Indies

## Abstract

The loss of regulating agents such as parasites is among the most important changes in biotic interactions experienced by populations established in newly colonized areas. Under a relaxed parasite pressure, individuals investing less in costly immune mechanisms might experience a selective advantage and become successful colonizers as they re‐allocate resources to other fitness‐related traits. Accordingly, a refinement of the evolution of increased competitive ability (EICA) hypothesis proposed that immunity of invasive populations has evolved toward a reduced investment in innate immunity, the most costly component of immunity, and an increased humoral immunity that is less costly. Biogeographical approaches comparing populations between native and expansion ranges are particularly relevant in exploring this issue, but remain very scarce. We conducted a biogeographical comparison between populations of Spectacled Thrush (*Turdus nudigenis*) from the native area (South America) and from the expansion range (Caribbean islands). First, we compared haemosporidian prevalence and circulating haptoglobin (an acute‐phase protein produced during inflammation). Second, we challenged captive birds from both ranges with *Escherichia coli* lipopolysaccharides (*LPS*) and measured postchallenge haptoglobin production and body mass change. Birds from the expansion range showed lower haemosporidian prevalence and lower levels of haptoglobin than birds from the native range. In addition, the inflammation elicited by LPS injection and its associated cost in terms of body mass loss were lower in birds from the expansion range than in birds from the native range. In accordance with the enemy release hypothesis, our results suggest that range expansion is associated with a reduced infection risk. Our study also supports the hypothesis that individuals from newly established populations have evolved mechanisms to dampen the inflammatory response and are in accordance with one prediction of the refined EICA hypothesis, proposed to understand biological invasions.

## 1 Introduction

Changes in species range have crucial effects on biodiversity at local, regional and continental scales (Brown, 1995). Whatever their proximal cause, species range expansion and colonization of new areas have attracted much attention as invasive species challenge the stability/resistance of communities, can disrupt ecosystem functioning, and may have associated negative ecological and economic effects (Levine, 2008). Identifying which traits characterize successful colonization of introduced species (Duckworth & Badyaev, 2007; Elton, 1958; Jeschke & Strayer, 2005) and conditions accompanying their persistence and spread could be particularly important to predict risks of biological invasions (Garcia‐Ramos & Rodriguez, 2002; van Kleunen, Weber, & Fischer, 2010; Kolar & Lodge, 2001; Parker et al., 2013).

Species range expansions offer relevant opportunities to investigate consequences of marked shifts in selection, as colonizing organisms have to face ecological pressures that contrast with those prevailing in their native range. The loss of natural enemies, such as parasites, is among the most important changes in biotic interactions experienced by spreading populations (Mitchell & Power, 2003; Torchin, Lafferty, Dobson, McKenzie, & Kuris, 2003). Several studies have described reduced parasite diversity and prevalence in invading host populations (Mitchell & Power, 2003; Torchin et al., 2003; Tsai & Manos, 2010; White & Perkins, 2012) and the loss of parasites from their native range (Hatcher & Dunn, 2011; Marzal et al., 2011), as well as the failure for local parasites to infect or to heavily exploit these new hosts (Cornet, Sorci, & Moret, 2010; Lee, Martin, & Wikelski, 2005). These observations provide support to the enemy release hypothesis (ERH) proposed as an explanation for the success of biological invasions, stating that exotic species become successfully established because they are freed from organisms that regulate them (Keane & Crawley, 2002). This hypothesis leads to predict that protective functions are of declining importance in newly colonized areas. Following an evolutionary perspective, the evolution of increased competitive ability (EICA) hypothesis (Blossey & Notzold, 1995) predicts changes in defense mechanisms with successful invaders being those that, under a relaxed parasite pressure, invest more to fitness‐related traits (e.g., reproductive activity, growth) or to dispersal ability, and less to defense mechanisms (Bossdorf et al., 2005; Horrocks, Matson, & Tieleman, 2011; Martin, Alam, Imboma, & Liebl, 2010; White & Perkins, 2012).

Immunity, which is probably the most crucial defense that hosts have evolved to fend off parasites, is costly for individuals (Eraud, Duriez, Chastel, & Faivre, 2005; Eraud, Jacquet, & Faivre, 2009; Graham, 2002; Martin, Scheuerlein, & Wikelski, 2003). Thus, the expression of immune defenses is constrained by their associated costs (Schmid‐Hempel, 2003; Viney, Riley, & Buchanan, 2005). In cases of new range colonization, where co‐evolutionary processes between hosts and pathogens are not finely adjusted, immune activation may be more costly than beneficial. Indeed, local parasites may be unable to exploit hosts, and the colonizing host may elicit an inappropriate and too strong response that would be at the origin of unnecessary metabolic costs and/or autoimmunity (White & Perkins, 2012). As a consequence, investment in immunity may shift toward a new and lower optimal level for host fitness (Horrocks et al., 2011) in non‐native ranges. Following this view, Lee & Klasing (Lee & Klasing, 2004) proposed that invasive vertebrate hosts should express a dampened systemic inflammatory response. Indeed, inflammation is among the most energetically and nutritionally costly component of vertebrate immunity, and because of its nonspecificity, it may inflict collateral damage to the host (Halliwell, 2006; Sorci & Faivre, 2009) with negative consequences on fitness (Belloni et al., 2014; Graham et al., 2010; Guerreiro et al., 2012). In addition, Lee and Klasing (2004) proposed that invasive populations should express an enhanced humoral response, because this antibody‐mediated immune component is less costly to produce.

The framework proposed by Lee and Klasing (2004) is a refinement of the EICA hypothesis and finds some support from studies that showed contrasted immunity between closely related bird species. Lee et al. (2005) reported a reduced Th1 immune response to inflammatory challenges in a highly invasive species when compared to a much less invasive related species, with negative effects of immune activation observed only in the less invasive species. In addition, Martin et al. (2010) compared an introduced bird species with a native and closely related one and observed a reduced inflammatory response and no body mass reduction in the introduced species. Biogeographical approaches, comparing intraspecific populations (*i.e.,* invasive *vs* native), could provide the most valuable insights. As a rare case study, Llewellyn, Thompson, Brown, Phillips, and Shine (2012) showed that Australian cane toads (*Rhinella marina*) close to the invasion front have a dampened immune response compared to individuals from long‐established populations, and a more recent common garden work on the same system found a less clear pattern, despite substantial changes in immune components have been observed (Brown, Phillips, Dubey, & Shine, 2015). Despite this handful of studies, and as recently emphasized by White & Perkins (White & Perkins, 2012), there is currently little empirical evidence on the ecological immunology of invasions, especially from the most powerful approaches that are biogeographical studies. To our knowledge, no experimental study following a biogeographical approach compares the inflammatory response between native and non‐native populations of the same species.

Here, we followed both descriptive and experimental biogeographical approaches to compare prevalence of blood parasites (*Plasmodium* and *Haemoproteus* genus), and inflammatory components among several populations of the Spectacled Thrush (*Turdus nudigenis*). This species is native to northern South America and southern Lesser Antilles and has steadily colonized Caribbean islands toward the north over the last century. First observations of the Spectacled Thrush in Saint Lucia and Martinique occurred in the early 1950s (Pinchon, 1963). The species remained scarce during several decades and started to spread over these two islands early in the 1990 (G. Tayalay & L. John, pers. comm.). Now, the expansion phase is continuing on both islands. More to the north, Dominica was colonized in the 1980s, and the Spectacled Thrush is still patchily distributed with limited population sizes (A. James & B. JNO Baptiste, pers. comm.). Guadeloupe is the northern edge of the present distribution area, where the Spectacled Thrush started to breed in this island in 1997, but is still rare today (Levesque, Duzont, & Ramshaï, 2005).

As patterns of reduced biodiversity on island (Losos & Ricklefs, 2009) are also observed for parasites (Wikelski, Foufopoulos, Vargas, & Snell, 2004), cases of potential hosts expanding their ranges on islands offer reliable conditions to observe reduced parasite pressure and to test the conceptual framework proposed by the EICA hypothesis and its refinement focused on immunity. Accordingly, we expected lower parasite prevalence in spreading populations than in native ones, lower levels of current inflammation, weaker responses of individuals exposed to an inflammatory stimulus, and lower associated costs in colonizing populations.

## 2 Materials and Methods

### 2.1 Bird capture and study sites

**Figure 1:**
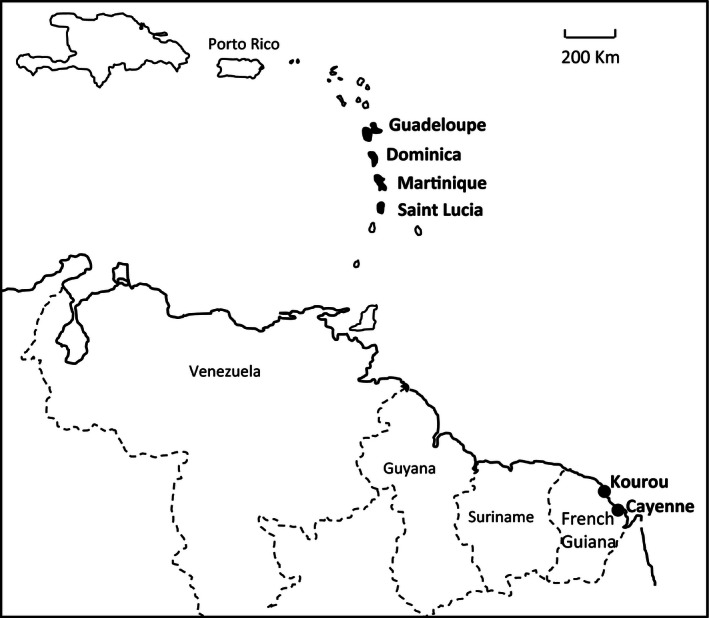
Map showing the location of six sites. Cayenne (4.56 N, 52.19W) and Kourou (5.09N, 52.39W) are into the native range of the Spectacled Thrush, whereas Saint Lucia (14.01N, 60.59W), Martinique (14.36N, 61.04W), Dominica (15.18N, 61.22W), and Guadeloupe (16.14N, 61.32W) have been recently colonized. Sampled sites are indicated in black

Bird capture was conducted between March 2011 and March 2013 at six sites (hereafter named populations) during the dry seasons: Kourou and Cayenne (French Guiana), Saint Lucia, Martinique, Dominica, and Guadeloupe (Figure 1). Kourou and Cayenne are into the native range of the Spectacled Thrush, while the four other sites have been colonized more or less recently by this species. Birds were captured using mist nets, weighed (±0.1 g.), and ringed with a numbered leg band. In Kourou, Cayenne, and Martinique, birds were then randomly assigned to descriptive or experimental studies. Because of logistic constraints in Saint Lucia and Dominica, and because of the small number of birds sampled in Guadeloupe (where abundance is low), only the descriptive study was carried out in these sites.

### 2.2 General procedure of the descriptive and experimental approaches

To achieve the descriptive study, we collected blood samples (100–250 μl) from the brachial vein using sterile needles and heparinized capillary tubes. Twenty microliters were immediately stored in 500 μl of Queen's lysis buffer (Seutin, White, & Boag, 1991) for molecular identification of sex and haemosporidian detection, and the remaining blood was kept into a cool box until centrifugation. Birds were immediately released after blood sampling. Blood was centrifuged (1900 g, 15 min, 4°C) within few hours after collection, and plasma was stored at −20°C until transport on ice to France. Finally, samples still frozen at arrival were stored at −80°C until analysis.

For the experimental study, captured birds were placed in cloth cages with water and food and held in capture sites until taken to sites where experiment was conducted. Birds were housed outdoor in cloth cages (height: 0.75 m., diameter 0.50 m). Housing facilities corresponded to isolated places sheltered from rain, direct sunlight, and predators (covered tropical greenhouse, or cowl). Universal softbill food, mealworms, mangoes, and water were provided daily *ad libitum*. After 2 days in captivity (H0), birds were weighed, bled as above described, and randomly treated with lipopolysaccharides from *Escherichia coli* (LPS; Serotype O55:B5; Sigma) to stimulate an inflammatory response, or with phosphate‐buffered saline (PBS) for control. LPS diluted in 200 μl of PBS (0.2 mg/ml) were intraperitoneally injected. Control birds received the same amount of PBS. LPS are recognized by the vertebrate immune system and induce a systemic inflammatory response characterized by a rapid delivery of effectors (Janeway & Medzhitov, 2002; Sorci & Faivre, 2009). To provide sterile injection at bleeding sites, the sites were carefully swabbed with 70% ethanol, and injection and blood sampling were carried out after air‐drying. Sixteen hours postinjection (H16), blood was collected from the brachial vein that has not been bled to obtain the pretreatment (H0) blood sample, and birds were released in their capture sites. Change in circulating haptoglobin within 16 hr postinjection was used to assess response of birds to treatments (LPS injection or control). Sixteen hours seemed appropriate to assess haptoglobin concentration as it tended to peak at this time in an exploratory work we did before this study (not shown). Blood was prepared and transported in the conditions described above before laboratory analyses. H0 took place at dusk, and birds were immediately placed back into their cage so that they had no access to food overnight and were not stimulated to eat the next morning because food was not renewed before H16. Thirty‐four birds from Cayenne (15 PBS and 19 LPS), 20 from Kourou (10 PBS and 10 LPS), and 40 from Martinique (20 PBS and 20 LPS) were included in the experiment. Haptoglobin was not assessed for four H0 plasma samples that we failed to conserve after centrifugation.

### 2.3 Sex determination and parasite detection

To determine sex, DNA was extracted from samples with a standard phenol–chloroform protocol (modified from Hillis, Mortitz, & Mable, 1996) after a first step of digestion with proteinase K (56°C overnight). Molecular sexing was performed following Fridolfsson and Ellegren (1999). In order to detect the presence of malaria parasites (*Plasmodium sp* and *Haemoproteus sp*), we amplified a 524‐bp fragment of the parasite mitochondrial cytochrome b (cyt*‐b*) gene, following the nested PCR protocol described by Waldenstrom, Bensch, Hasselquist, and Ostman (2004). The first and second rounds of PCR were, respectively, performed in 10 and 25 μl, and negative controls (water) were included to detect false positive (contaminations). PCR products were visualized on 2% agarose gels, and parasite presence was detected by comparison with a positive control (sample with known infection). Individuals presenting a negative signal of parasite DNA amplification were re‐analyzed to confirm their negative status.

### 2.4 Haptoglobin assessment

Haptoglobin is an acute‐phase protein that is produced from the liver during an inflammatory response and that has been detected in several bird immunology studies (Cellier‐Holzem, Esparza‐Salas, Garnier, & Sorci, 2010; Martin et al., 2010; Millet, Bennett, Lee, Hau, & Klasing, 2007). We quantified this protein to assess baseline levels (descriptive approach) and to assess response to the immune challenge (experimental approach). Circulating haptoglobin was assessed using a commercial assay kit (TP‐801; Tridelta Development Ltd., Ireland) following the manufacturer's instructions. First, 7.5 μl of each plasma sample or standard was randomly deposited on flat‐bottomed 96‐well plates. One hundred microliter of a solution of hemoglobin and 140 μL of a chromogen solution were added in each well. Plates were then agitated and let to incubate for 5 min at room temperature. Then, plates were read at 630 nm. Standard curves were obtained from serial dilutions of an initial standard beginning at 2.5 mg/ml. They included five dilutions to 0.075 mg/ml. Intra‐assay variation based on eight to 14 duplicates was low (3.311 ± 1.061%) as well as interplate variation based on five samples repeated over plates (4.032 ± 0.506%).

### 2.5 Statistical analysis

Potential variation of parasite prevalence between native and newly colonized areas was tested using a generalized linear model (GLM‐0) with a binomial distribution of errors and a logit link function. The dependent variable was the presence/absence of blood parasite, and the full model comprised the following explanatory variables: area (colonized *vs* native) and population (*i.e.,* Kourou, Cayenne, St Lucia, Martinique, Dominica, or Guadeloupe, with population nested in area). Sex and body mass of individuals were included as covariates, as they may also affect the infection risk. Then, we tested whether the baseline expression of inflammation varied between native and colonized areas using a generalized linear model (GLM‐1). The dependent variable was the haptoglobin concentration measured at capture, and the explanatory variables were the same as in GLM‐0 plus the parasitic status of individuals (infected vs noninfected).

With the experimental approach, we aimed at testing for potential differences in inflammatory response to immune stimulation and in phenotypic costs associated with inflammation between colonizing and native individuals. To assess the impact of captivity on individuals, we compared (GLM‐2) circulating haptoglobin concentration (measured at capture or at H0; dependent variable) between birds kept in captivity and birds used in the descriptive approach. As previously, population, sex, and body mass (recorded at capture or at H0) were also included in the model. In addition, we tested whether captivity affected individual condition by comparing body mass measured at capture and mass measured at H0 (*t*‐test for paired samples). Finally, we used GLMs (respectively, GLM‐3 and GLM‐4) to compare (1) variations in haptoglobin concentrations (difference between measures at H16 and H0, dependent variable) and (2) variations of body mass (difference between measures at H16 and H0, dependent variable) between native and colonizing individuals, consecutively to an immune activation. We included treatment (LPS vs PBS), population, variation of body mass between H16 and H0 (only for GLM‐3), sex, haptoglobin concentration at H0 (only for GLM‐3), parasitic status, and the interactions between treatment and population and between treatment and variation of body mass (only for GLM‐3) as explanatory variables. For each model, nonsignificant variables were removed following a stepwise procedure. All analyses were conducted using R version 3.2.3 (R Development Core Team 2015).

## 3 Results

### 3.1 Malaria prevalence and baseline haptoglobin levels

**Figure 2:**
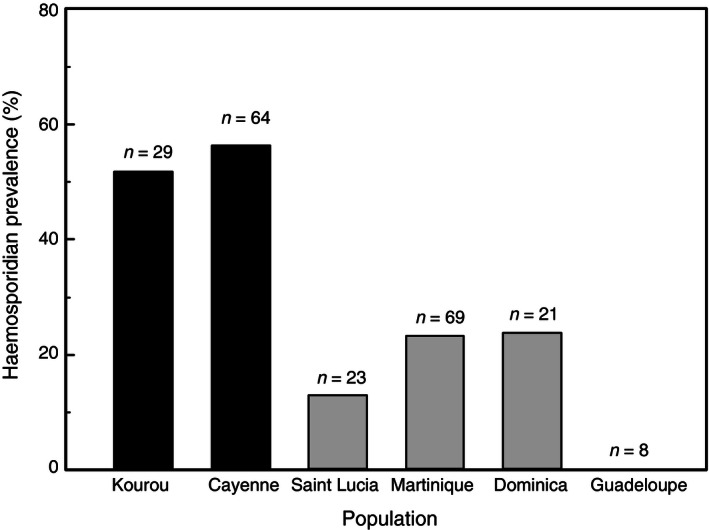
Prevalence of haemosporidian in two populations from the native range (Kourou and Cayenne) and four populations from the newly colonized range (Saint Lucia, Martinique, Dominica, and Guadeloupe). Sample size is added above bar chart

Results of GLM‐0 showed a significant effect of area (*χ*² = 29.78, *p *<* *.0001) and sex (*χ*² = 6.98, *p *=* *.008) with a higher prevalence of blood parasites in native populations than in colonizing populations of spectacled thrushes and a higher prevalence in males (Figure 2). Conversely, neither population nor body mass at capture affected prevalence significantly within the two areas.

**Figure 3:**
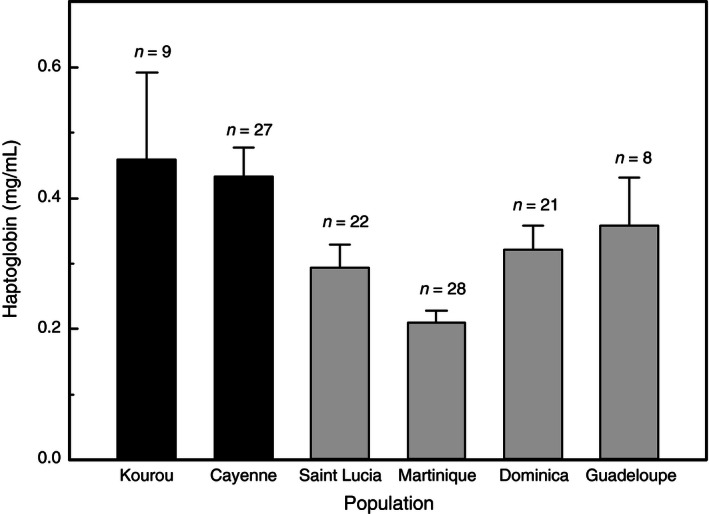
Baseline concentrations of haptoglobin (mean ± SE) in two populations from the native range (Kourou and Cayenne) and four populations from the newly colonized range (Saint Lucia, Martinique, Dominica, and Guadeloupe). Sample size is added above bar chart

Results of GLM‐1 showed significantly higher levels of haptoglobin in native populations than in colonizing ones (*F*_1,110_ = 5.470, *p *=* *.021, Figure 3). Body mass at capture significantly influenced the measured concentration with heaviest individuals showing higher level of circulating haptoglobin (*F*_1,110_ = 12.430, *p *<* *.001). Population, parasitic status (*F*_1,110_ = 0.087, *p *=* *.769), and sex (*F*_1,110_ = 1.150, *p *=* *.286) did not significantly affect the baseline level of haptoglobin. We also found that body mass differed between native and expanding areas (*F*_1,110_ = 33.918, *p *<* *.001) as well as between populations (*F*_4,110_ = 5.769, *p *<* *.001).

### 3.2 Experimental activation of inflammation and associated costs

**Figure 4:**
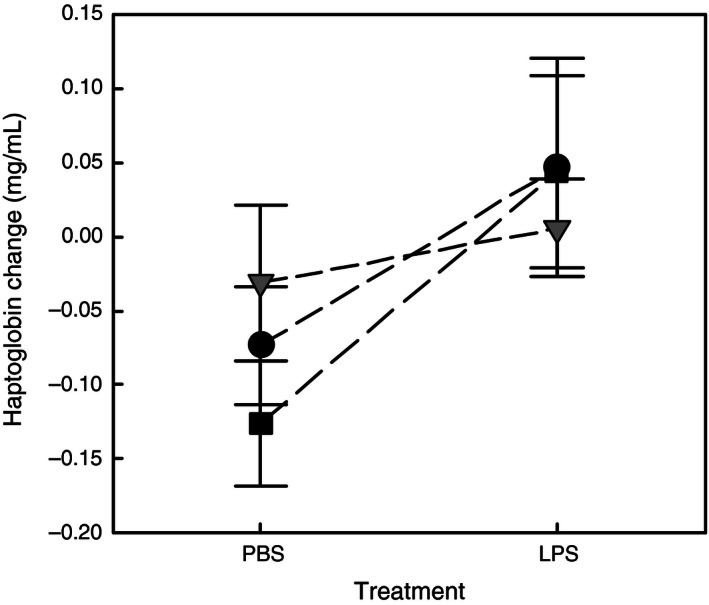
Change of circulating haptoglobin concentration (mean ± SE) between injection and 16 hr postinjection of LPS (immune stimulation) or PBS (control). Black squares, black circles, and gray triangles depict birds from Cayenne (PBS,* n* = 15; LPS,* n* = 16), Kourou (PBS,* n* = 10; LPS,* n* = 10), and Martinique (PBS,* n* = 20; LPS,* n* = 19), respectively

Levels of haptoglobin quantified for housed birds at H0 (*i.e.,* after 2 days in captivity and immediately before the experimental activation of inflammation) were higher than levels measured at capture for birds that have not been housed (GLM‐2; *F*_1,146_ = 21.855, *p *<* *.001), showing that captivity enhanced production of this molecule. As previously, levels of haptoglobin were significantly affected by body mass (GLM‐2; *F*_1,146_ = 19.219, *p *<* *.001), but not by population or sex (all *ps >* .278). Experimental treatment significantly affected change in haptoglobin measured 16 hr postinjection (GLM‐3, Table 1). The response to the immune challenge, illustrated by the difference in haptoglobin production trajectories between LPS and PBS groups, depended on the population as witnessed by a significant interaction between population and treatment (Table 1, Figure 4). Indeed, birds from the two native populations (French Guiana) tended to show a stronger decrease of haptoglobin after injection of PBS and a higher increase (even slight) after LPS injection than birds from the colonized area (Martinique), leading to a larger difference of circulating haptoglobin between PBS and LPS groups in native birds than in colonizing birds (Figure 4). In addition, changes in circulating haptoglobin within 16 hr postinjection were negatively associated with initial values measured immediately before injection (H0) and positively with body mass changes in the same time (Table 1). Because initial levels of haptoglobin did not differ between experimental groups within each population (*t*‐tests, all *ps >* .606), any bias due to initial differences between groups instead of the treatment factors can be discarded. Finally, change in haptoglobin was not significantly affected by sex or parasitic status (all *ps >* .200).

**Table 1:** Analysis of factors affecting haptoglobin and body mass changes (H16‐H0 measures) consecutively to an immune activation. Only final models, resulting from a backward selection of significant variables, are reported

		Estimate	SE	*df*	*F*	*p*‐Value
GLM‐3						
Haptoglobin change						
Treatment	LPS	0.189	.048	1	15.314	<.001
Haptoglobin at H0		−0.601	.059	1	103.067	<.001
Body mass change		0.032	.009	1	11.998	<.001
Population	Kourou	0.024	.053	2	1.205	.305 (NS)
	Martinique	0.067	.044			
Treatment × population	LPS: Kourou	0.028	.074	2	6.185	.003
	LPS: Martinique	−0.188	.064			
GLM‐4						
Body mass change						
Treatment	LPS	−1.394	.512	1	7.403	.008
Body mass at H0		−0.108	.038	1	7.987	.005
Population	Kourou	−0.122	.615	2	1.636	.201 (NS)
	Martinique	−0.954	.576			
Treatment × population	LPS: Kourou	−0.231	.838	2	3.712	.028
	LPS: Martinique	1.594	.695			

**Figure 5:**
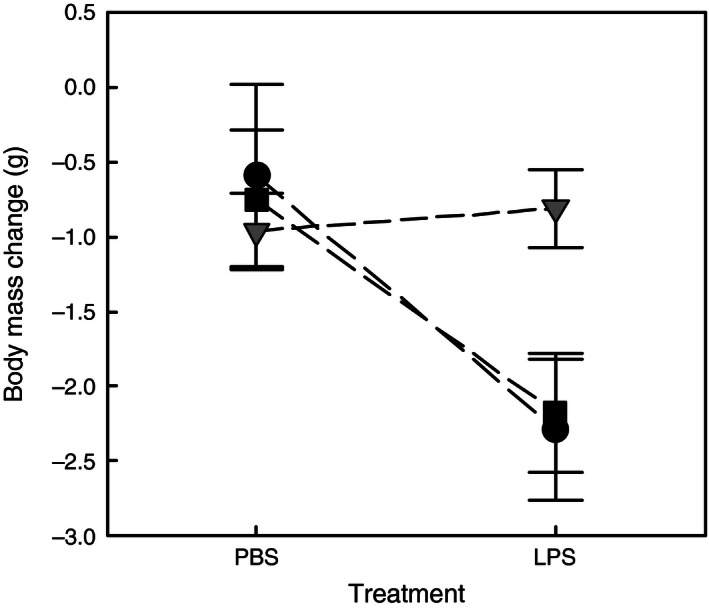
Change of body mass (mean ± SE) between injection and 16 hr postinjection of LPS (immune stimulation) or PBS (control). Black squares, black circles, and gray triangles depict birds from Cayenne (PBS,* n* = 15; LPS,* n* = 19), Kourou (PBS,* n* = 10; LPS,* n* = 10), and Martinique (PBS,* n* = 20; LPS,* n* = 20), respectively

In contrast to haptoglobin, body mass was not significantly affected by captivity although it tended to increase after bird caging (paired *t*‐test = 1.914, *p *=* *.058). Body mass change was influenced by experimental treatments as LPS birds lost more weight than PBS (control) injected birds (GLM‐4; Table 1, Figure 5). However, as for circulating haptoglobin, there was a significant interaction between population and treatment (Table 1), with LPS birds loosing clearly more body mass than PBS ones in French Guiana, whereas change of body mass appeared quite similar between the two treatments in birds from Martinique (Figure 5). As for changes in circulating haptoglobin which depended on initial level, body mass loss depended on the initial body mass measured at injection time (Table 1), as it was higher for heavier birds at H0, but was not significantly affected by sex. Because initial body mass did not differ between experimental groups within each population (*t*‐tests, all *ps >* .590), any bias due to initial differences between groups instead of the treatment factors can be discarded.

## 4 Discussion

To test the downward expression of the inflammatory process in ranges newly colonized by expanding species, we conducted a biogeographical approach and compared baseline levels of haptoglobin, and haptoglobin production after an experimental inflammatory stimulus in spectacled thrushes between native and expanding populations. Our results provide evidence for a reduced parasite pressure and a reduced baseline production of haptoglobin in the expanding range. In addition, individuals from this range produced a lower inflammatory response and lost less body mass than control birds when challenged with LPS.

### 4.1 Malaria prevalence and baseline haptoglobin

Our results provide some support for the still‐debated ERH. Parasite prevalence was of the same order of magnitude in the two Guyanese native populations (52% and 56%) and is similar to prevalence recorded in central Brazil in the Pale‐breasted thrush (*Turdus leucomelas*; 55%), a turdid species sharing the same habitats than the Spectacled Thrush (Belo, Pinheiro, Reis, Ricklefs, & Braga, 2011). In Caribbean populations of Spectacled Thrush, parasite prevalence (0–23%) was clearly lower or in the same order than the lower overall prevalence observed in four common Antillean birds studied in nine islands, including the four used in this study (Fallon, Bermingham, & Ricklefs, 2003). Indeed, Fallon et al. (2003) found an overall prevalence of 52% in the Lesser Antillean bullfinch (*Loxigilla noctis*), 45% in the Bananaquit (*Coereba flaveola*), 35% in the Black‐faced grassquit (*Tiaris bicolor*), and 21% in the Black‐whiskered vireo (*Vireo altiloquus),* with no or little effect of the island on the distribution of overall prevalence across islands. In addition, a study conducted in Guadeloupe and Dominica on other thrush species, the Forest thrush (*Cichlherminia lherminieri*), showed prevalence of 62% and 78%, respectively (Arnoux, 2012). The reduced prevalence observed here suggests that the Spectacled Thrush remains an uncommon host for haemosporidans, even in islands (such as Martinique) where it has now spread. Additional investigation should also explore parasite intensity to better depict the contrast between native and non‐native populations.

Two other interesting points can be noted. First, apart from Guadeloupe, prevalence observed here is higher than that recently reported in the Spectacled Thrush by Ricklefs, Gray, Latta, and Svensson‐Coelho (2011) in three Caribbean islands (Saint Lucia: 0%, Saint Vincent: 0%, and Grenada: 7%). An effect of sampling season is unlikely because (1) our sampling period is the same than in Ricklefs et al. (2011) and (2) haemosporidian prevalence seemed not vary seasonally, as shown in two other Caribbean islands (Porto Rico and Saint Lucia, Fallon, Ricklefs, Latta, & Bermingham, 2004). A long‐term effect is more likely as Ricklefs et al. (2011) used samples mostly collected in early 1990s and 2000s, a timescale compatible with observable changes in prevalence (Fallon et al., 2004). At Saint Lucia, sampling by Ricklefs et al. (2011) occurred sooner after colonization of the islands than our sampling. Several case studies demonstrated a transient parasite release in expanding species (Gendron, Marcogliese, & Thomas, 2012; Hajek & Tobin, 2011; Phillips et al., 2010). Second, while population did not significantly affect haemosporidian prevalence in our study, the absence of parasite in Guadeloupe is noteworthy. Indeed, if cautious interpretation is required because of the small sample size, this observation may illustrate the expected preponderance of uninfected hosts close to the expansion front (Phillips et al., 2010; White & Perkins, 2012). Overall, our results suggest a reduced parasite pressure in spectacled thrushes that have colonized the Lesser Antilles, and let us expect dampened inflammatory responses in this part of the species' range.

The baseline levels of circulating haptoglobin measured here fell in the range of those assessed by Matson (2006) in similar conditions (*e.g.,* upon capture) in nine passerine genera from islands or continental masses (0.09–0.45 mg/L). We detected a clear difference between the two native and the spreading populations, suggesting a lower inflammatory status of colonizing birds. Higher levels of circulating haptoglobin in birds from native populations might result from a stronger innate immunity or from an ongoing reaction to infections due to a higher parasitic risk than in non‐native birds. Our results provide some arguments to rule out the second explanation because circulating haptoglobin was not associated with parasitic status of birds, suggesting that individuals were under chronic infection stages. Of course, an acute response to other pathogens than haemosporidian might explain the higher levels observed in native populations, but our results may also suggest a reduced investment in inflammation in spectacled thrushes from spreading populations.

Additionally, because body mass differed between native and colonizing populations, we may hypothesize that the area (native vs colonizing) effect is due to this body mass contrast. This seems unlikely because body mass differed also between populations (whatever the area) while haptoglobin level did not. However, our results underline that body mass is another life history trait that discriminated colonizing from native populations, and body mass contrast might be among proximal factors that govern baseline levels of haptoglobin.

### 4.2 Experimental activation of inflammation

Experiments of immune activation have been conducted on captive birds, and we first observed that captivity increased levels of haptoglobin. Captivity imposes behavioral and physiological stresses (Dickens, Earle, & Romero, 2009; Mason, 2010) that subtly interact with and affect immunity (Ewenson, Zann, & Flannery, 2001; Kuhlman & Martin, 2010; Martin, 2009; Martin, Brace, Urban, Coon, & Liebl, 2012). A shift toward a pro‐inflammatory status has been recently found in house sparrows held for several weeks (Martin, Kidd, Liebl, & Coon, 2011). The duration of the stressor is a key component of the stress‐induced alteration of immunity (Martin, 2009), and our results suggest that inflammation was already processing within a short time after caging. Further investigation, and especially glucocorticoid assessment, should help to elucidate the mechanisms involved in this activation, but an effect through body mass change seems unlikely because captivity did not induce significant body mass change in our case study.

The experimental stimulation of inflammation showed a treatment effect with a decrease of circulating haptoglobin levels in control birds while they slightly increased or remained unchanged in LPS‐challenged birds. As mentioned above, there is a link between stress induced by captivity and inflammation, and we may hypothesize that stress triggered by captivity became steadily dampened in the short term after caging as suggested by the decrease of haptoglobin production observed in control birds. As a consequence, the response to the immune challenge (*i.e.,* LPS treatment) can only be assessed by the difference in haptoglobin production trajectories between LPS and PBS groups, as absolute levels of haptoglobin in LPS group may result from both a decline following recovery from the stress of captivity and an increase due to the immune challenge. Haptoglobin contributes to protect host from microbicidal reaction elicited by infection (Gabay & Kushner, 1999), and our results showed its production in response to antigen challenge as reported in previous bird studies assessing this inflammatory marker (van de Crommenacker et al., 2010; Martin et al., 2010; Millet et al., 2007). Importantly, our experimental findings support one prediction of the framework proposed by Lee and Klasing (2004), that is, a reduced inflammatory response in spreading populations. Indeed, the significant interaction between treatment and population suggests a dampened production of haptoglobin in response to LPS challenge in non‐native birds from Martinique compared to Guyanese birds. Interestingly, Spectacled Thrush population in Martinique is currently at the third stage mentioned for successful invasions (*i.e.,* the range expansion or spread stage characterized by a sharp increase of abundance; Blackburn et al., 2011), and differential selection between the native and the newly colonized areas may become important at this stage (White & Perkins, 2012). However, our results support both EICA, that is, globally reduced immune defenses and reallocation of resources to other fitness‐related traits, and refined EICA, that is, decrease of costly inflammatory reactions and enhancement of adaptive immunity. Therefore, further work is required to investigate more completely the hypothesis of Lee and Klasing (2004) by testing an enhanced adaptive immunity in non‐native populations.

To our knowledge, no experimental study following a biogeographical approach compared inflammatory markers between native and non‐native populations of the same species. Our results are not in the line of those recently found by Brown et al. (2015), who showed higher levels of inflammatory components in cane toads originating from the invasion front in Australia. However, they are consistent with those rising from the few comparisons between closely related species of sparrows. Indeed, Lee, Martin, Hasselquist, Ricklefs, and Wikelski (2006) compared American populations of the highly invasive house sparrow to its less successful relative, the tree sparrow (*Passer montanus*), and found a weaker local inflammatory response in the invasive species. In Kenya, Martin et al. (2010) showed that challenged non‐native house sparrows produced fewer haptoglobin than native rufous sparrows (*Passer rufocinctus*).

Alternatively, the comparison of haptoglobin change could suggest that the expanding populations did not reduce the stress due to captivity while the native ones did. Even if this hypothesis is not supported by results on body mass change, it cannot be ruled out, and again, investigation including stress hormone assessment should help to discuss our results further.

Body mass did not change after 2 days in captivity suggesting that food intake was not affected by captivity. However, bird handling for experiment probably induced loss of mass because all experimental groups showed the same trend. Importantly, the overall pattern of body mass change suggests that inflammatory response was more costly in native populations than in non‐native Caribbean birds. Indeed, antigen‐challenged individuals showed a greater decrease in body mass than control birds, but beyond this global negative effect of LPS that is commonly described in vertebrates (Belloni et al., 2014; Lochmiller & Deerenberg, 2000), non‐native spectacled thrushes from Martinique seemed insensitive to the activation of inflammation in terms of mass loss. In birds from native populations, the stronger inflammatory response might have incurred body mass loss through anorexia, that is a classical component of the sickness behavior (Hart, 1988; Kyriazakis, Tolkamp, & Hutchings, 1998; Owen‐Ashley & Wingfield, 2006), or through elevation of metabolic rate that induces energy consumption (Lochmiller & Deerenberg, 2000). We did not assess these behavioral and physiological consequences of inflammation, and we cannot speculate on mechanisms that caused body mass loss in our case study. However, because the most part of the 16 hr postinjection elapsed during nighttime, an effect driven by metabolic costs seems more likely. To our knowledge, only one biogeographical study evidenced experimentally an attenuated cost of inflammatory response in colonizing populations (Llewellyn et al., 2012). In this study, the elevation of metabolic rate in LPS‐challenged cane toad (*Rhinella marina*) was lower in individuals from the invasion front than in those from long‐established populations in Australia.

Finally, at first glance, the above interpretation on body mass change seems not supported by the positive association between haptoglobin change and body mass change (see GLM 3, Table 1). Again, mechanistic investigation should deserve attention to disentangle factors that control immune response in expanding populations.

Our descriptive and experimental results together provide support for both the ERH and the hypothesis that investment in the inflammatory response is reduced in non‐native populations that recently colonized new ranges. They suggest that expanding birds displaying reduced inflammatory processes might have experienced a better fitness in newly colonized areas where they were at least partially freed from parasites able to exploit them. Given the link existing between immunity and dispersal ability (Brown and Shine 2014), a partially alternative hypothesis is that individuals dispersing from native areas to start new expanding populations were mostly endowed with a reduced inflammatory component. Further investigations are needed to better depict the infectious status and intrinsic immune profile of expanding populations, and integrative studies should examine potential trade‐offs between immunity and other key life history traits at different steps of the expansion process. The study of antibody‐mediated immunity would be particularly relevant to test more completely the refined EICA hypothesis. In addition, the investigations of fitness‐related traits, for example, reproductive output and/or survival, would be important to explore whether the resources saved by a reduced allocation to immunity are beneficial to expanding birds. Such integrative studies, combining descriptive and experimental approaches, will provide a better understanding of the role of host–parasite interactions and host immunity in species range expansion and in the success of biological invasions.

## Data archiving

Data will be archived in an appropriate public archive (DRYAD).

## Conflict of Interest

None declared.
